# The effect of attire attractiveness on students’ perception of their teachers

**DOI:** 10.3389/fpsyg.2022.1059631

**Published:** 2023-01-09

**Authors:** Marius Marici, Remus Runcan, Iasmina Iosim, Alexandra Haisan

**Affiliations:** ^1^Department of Educational Sciences, Ștefan cel Mare University of Suceava, Suceava, Romania; ^2^Department of Pedagogy, Psychology and Social Work, Aurel Vlaicu University of Arad, Arad, Romania; ^3^The King Michael I University of Life Sciences, Timișoara, Romania

**Keywords:** attire attractiveness, teachers, clothing, children, personality traits

## Abstract

**Introduction:**

*Generally, people do judge a book by its cover*. *The purpose* of this research is to investigate the effect of teachers’ attire on students’ perception of 34 psychological dimensions.

**Methods:**

*The research* is an experiment, with self-reported data, in groups, based on a questionnaire. The participants were 173 students (M_age_ = 12.16, SD = 1.74) from Suceava, Romania. Two groups of students were asked to listen to a sample lesson of a therapeutic story, narrated by a teacher. One group was given a picture of the attractive teacher and the other group a picture of the unattractive teacher, and were told that the teacher who is narrating is the teacher in the picture. After listening to the same story, the respondents had to answer a questionnaire about teachers’ personality and characteristics.

**Results:**

*The results* indicated that when the teacher is perceived as being more attractive, the students have a greater openness for school activities, the evaluation of the teacher’s personality is more positive, the evaluation of the teaching effort is more positive, students expect a higher grade, and the perceived age of the teacher is lower.

**Discussion:**

The article underlines the role of clothing in molding student’s perception and raises questions about dress codes in schools. Implications for school context are discussed.

## 1. Introduction

‘Clothing is one of the essential elements of human civilization.’ (Jayasooriya et al., 2020, p. 171). Research indicates that clothing is a significant form of nonverbal communication that affects the perceptions of others (Dixon, 2007). Few studies have investigated teacher dress in the classroom, and the field is less studied (Carr et al., 2009). Yet, the field represents ‘a fruitful area of research’ (Johnson et al., 2014, p. 21), which deserves all our attention.

Romania is a country where dress codes in schools are not mandatory, but schools can adopt such codes on their own decision. Generally, teachers do not have dress codes and they can dress according to general principles, written in local school regulations or required by school principals, or in most cases are free to decide what clothing to wear and when.

## 2. Effects of human attractiveness

Studies indicate that there is a general perception that *all that is beautiful is good*, meaning that when the external evaluation is positive *the internal evaluation* or traits will tend to be positive as well. One of the most surprising conclusions regarding clothing evaluation is that, when the perception of clothing attractiveness is high, this external evaluation influences the internal evaluation of the person, and this happens in the absence of certain evidence that the person possesses particular internal characteristics, with a positive valence. In other words, a logical inference is made, in which it is considered that, if an individual is perceived as being visually attractive, then she/he *must* be a person who possesses internal qualities worthy of appreciation. It is a stereotype, an error in interpretation: attractive individuals possess positive personality traits (Armstrong, 2013).

Research in the field of human attractiveness referred to more components: facial attractiveness (Nestor et al., 2010), physical attractiveness (Li et al., 2019), vocal attractiveness (Shang and Liu, 2022), hairstyle attractiveness (Mesko and Bereczkei, 2004), or attire attractiveness (Sebastian and Bristow, 2008). Studies also investigated the association between height and attractiveness (Shepperd and Strathman, 1989) or shoes and attractiveness (Morris et al., 2013).

More physically attractive people are perceived as being more *competent* than less attractive people (Jackson et al., 1995), more *intelligent* (Kanazawa and Kovar, 2004) and have more positive *traits* (Feingold, 1992). Physical attractiveness produces a stronger impact on *employment* than its lack (Hosoda et al., 2003). Even *jurors* are influenced by the physical attractiveness of the accused (Mazzella and Feingold, 1994). A physically attractive instructor is *easier to follow* than an unattractive instructor (Westfall et al., 2016). Students believe that a physically attractive instructor would succeed in motivating them more, than an unattractive one (Westfall et al., 2016). Adults tend to consider a deviant behavior, such as throwing a rock at a dog, as *less serious* if the behavior is performed by an attractive child. Physically unattractive children are perceived as more *lying* and unpleasant than attractive children in the same scenario (Dion, 1972).

Regarding facial attractiveness, in the case of children with facial deformities, mothers talk less to them and are *less loving* than mothers who have children with non-facial deformities (Field and Vega-Lahr, 1984; Allen et al., 1990). People cooperate better with financial partners with attractive faces (Pandey and Zayas, 2021) and facial attractiveness plays a significant role in economic decision making (Ma et al., 2017).

Vocal attractiveness also plays a role in decision making and scientists coined the term “beauty premium” to refer to the advantages gained by more attractive people (Shang et al., 2021). Vocal attractiveness can also predict agreeableness and conscientiousness in job performance (Degroot and Kluemper, 2007). Men with attractive voices earn more votes and more often elected than men with unattractive voices (Tigue et al., 2012). Voice pitch is related to male and female attractiveness in school setting (Gumelar and Gilipanda, 2019).

Women wearing high hills are more sexually attractive than flat shoes. High hills are considered supernormal stimulus and increase the femininity of gait and thus sexuality perception (Morris et al., 2013). Another study found that women in high hills are perceived as being more physically attractive, sexually appealing, more feminine, and of a higher status (Wade et al., 2022).

Shorter females are considered more attractive than taller females regardless of the height of males when it is about dating (Shepperd and Strathman, 1989). Another study showed on the contrary that the higher the height the more attractive the females are (Worth, 2004).

Females with long hair are considered more attractive than females in short hair and healthier by men, especially if women are less attractive. Long hair, as it is harder to care for, it is associated with high phenotypic and genetic quality (Mesko and Bereczkei, 2004).

Many of the attractiveness elements are studied together and they are statistically associated. For example, body attractiveness could include attire, height or hairstyle attractiveness, without clearly discriminating between types of attractiveness. Facial and vocal attractiveness are associated in the mind of the assessor with ‘traits indicative of sex hormone levels … in order to assess mate value’ (Shang and Liu, 2022, p. 1–2). Another study found that hairdressing has a large effect on face attractiveness (Mesko and Bereczkei, 2004), while facial attractiveness has an effect on clothing attractiveness, in women (Niimi and Yamada, 2020).

## 3. The influence of teachers’ attire attractiveness on students

The field of education sciences has dealt too little with this topic, and there is a lack of research regarding the impact of teachers’ dress on students (Harbin, 2018). However, this is the situation in the context in which there have been and are various educational discussions regarding the appropriate or decent dress for students and teachers (Kashem, 2019). According to the common sense, it would seem that clothing does not matter at all, but the actual research indicates that teachers’ attire really influences students’ personality and performance, and it does matter a lot.

Teachers attire plays a significant role in establishing a teacher’s *responsibility, status, authority, competence,* or *success* (Turner-Bowker, 2001; Johnson et al., 2007). Teachers’ attire communicates who the person is as *an individual* and as a *professional* (Carr et al., 2009). In addition, teachers are *role model* for students regarding the style of clothing (Workman and Freeburg, 2010).

Goebel and Cashen (1979) found that, in general, teachers who do not value physical appearance are perceived to be less *friendly*, less *organized*, are perceived to encourage less student interaction, and are less likely to influence their students’ performance. Thus, clothing attractiveness can influence others’ perceptions of *personality traits*. Clothing attractiveness is an element that provides information about personality traits, personal *habits* and *skills*, or even socioeconomic *class* (Johnson, 1982). Molloy (1988) found that teachers’ dress style significantly affects students’ *attitudes.* This research indicated that conservative dress had a substantial, positive effect on both classroom *discipline* and the students’ *work* and *habits.*

Rollman (1980) provided further evidence regarding the impact of teacher dress on students. He concluded that those who dressed conservatively were perceived as highly *organized*, while the casually dressed teachers were perceived as *friendlier* and more *flexible*. The conclusions of these studies are also reinforced by the study of Workman and Johnson (1994). The study showed that students’ perceptions of teacher attributes are affected by their attire. The sample consisted of students from the 4th, 7th, and 9th grades, 27 of them being males and 33 females. Participants were shown three photographs of a female model dressed in three types of clothing: casual, everyday style, conservative, and modern. By means of a Likert scale, their task was to indicate their perception of the personality traits of the presented models. The results indicated that different styles of dressing tend to provoke different perceptions and opinions: ordinary or everyday clothing was perceived by students as conveying *friendliness, fairness,* and *interest;* modern or elegant clothing conveyed the idea of *organization, understanding*, and *discipline;* conservative dress elicited perceptions of *discipline*, *knowledge*, and *skill.* Although differences in perceptions were observed at different ages, no different perceptions due to student gender were noted (Workman and Johnson, 1994). Other studies have found that formal clothes indicate *competence* and *authority*, while casual clothes indicate *closeness* and *friendliness* (Peluchette and Karl, 2007). Workman and Freeburg (2010), however, also identified three categories of clothing for teachers regarded as inappropriate clothing: casual clothing, sexually provocative clothing, and clothing that violate conventional norms (Freeburg and Workman, 2010).

Another study found that when instructors were more attractive, students achieved higher *academic performance* (Westfall et al., 2016). In addition, more attractive students are perceived by teachers to be more *self-confident* and to have better *leadership traits* (Clifford and Walster, 1973) than unattractive ones. Academic performance is influenced by teacher attire (Behling and Williams, 1991). Teachers’ attire influences children’s psychology such as: *learning ability*, *motivation*, *discipline*, *working style*, or *attitudes* (Freeburg et al., 2011).

## 4. Children’s perceptions and attire attractiveness

There is a line of research that has also investigated the effect of teachers’ attractiveness on pupils’ or students’ school/academic performance. Studies indicate that pupils or students interpret reality differently in accordance with teachers’ attire. Other studies found that children exhibit the same biases as adults do. Children attribute more positive traits to more attractive children and more negative traits to less attractive children. Attractive children are perceived as exhibiting *prosocial personality traits*, while unattractive children are perceived as exhibiting *antisocial personality traits* (Dion, 1972). Children are more inclined to choose more attractive children *as friends* over less attractive children (Byrnes, 1987).

One explanation for the emergence of such interpretations in adults and children is that individuals start from what they know and express themselves about what they do not know, which leads to generalization. Wright (1988) stated that for this negative bias to occur three conditions are necessary, namely: (1) What is observed it must be obvious and clear and this conveys the idea of information saturation. A child who is disabled is more visible if he/she is asked to stand, than if all sit in chairs. (2) The observed characteristic must be considered negative. For example, if we want to select a boy for the debating club and he is disabled, it will not matter as much compared to the case where the child is to be selected for the athletics team. (3) The context of the feature is vague and undefined. If, for example, children on a volleyball team were to decide about a disabled child to be part or not of their team, then they would be more negative about this as compared to the situation in which the context of the disability was clearer and they had information about the prosthesis that he was wearing, or about the context in which the accident occurred, or about the child’s actual performance in sports (Wright, 1988).

Research also tried to counteract the effect of favoring students based on attractiveness of clothing, and the education system has come up with solutions (Sampson, 2016). It is known that until the end of the 60s there was a need for dress codes. Students normally wore the best clothes they could afford and were also more motivated to groom themselves. However, in democracy and with the expansion of cultural influences, students’ clothing style has changed, coming under the jurisdiction of personal choice. Inevitably, teachers’ attire passed through the same changes. Dress codes were created to eliminate this chaos and restore order in classrooms, although there are studies which also advocate for the negative effects of dress codes in schools (Whitman, 2020).

## 5. The present research

Existing research focused on studying attractiveness by combining several variables, such as clothing, make-up, hair styling and facial expressions, which would make it more difficult to isolate the effects of all these variables individually (Lennon, 1990). Studies have indicated that facial attractiveness influences clothing attractiveness especially in women (Niimi and Yamada, 2020). Therefore, the present research investigated only clothing attractiveness, and isolated the effect of facial expression.

In general, little research has investigated the attractiveness of teachers and the link between students’ perception of their teachers attractiveness and the assessment of the actual teachers’ personality characteristics (Bonds-Raacke and Raacke, 2007).

The purpose of the present research is to investigate the students’ perception of their teachers’ attire as well as the students’ perception of the teachers’ personality and behaviors, in the context of an experimental research.

## 6. Research methodology

### 6.1. Participants

The research participants were 173 children from Suceava, from neighboring villages, aged between 9 and 14 years old (M = 12.16, SD = 1.74). Of these, 50% were in 4th grade, 10.1% were in 6th grade, 28.7% were in 7th grade and 11.2% were in 8th grade. In the study participated 84 boys and 89 girls. The participants (10 classes of students) were extracted from a total pool of 22 classes of students (each class with between 8 and 28 children) from 5 different schools in Suceava, which were chosen by availability. The participants belonged to schools where no dress codes were required neither for students nor for teachers.

### 6.2. Instruments

The data was collected on the basis of a self-reported questionnaire after the participants listened to an audio recording and viewed a photograph of the speaking teacher.

The present research used the following instruments:

#### 6.2.1. Perception of the teacher’s personality

The research used The Rokeach Value Survey (RVS) scale (Rokeach, 1973), especially The Instrumental Values subscale, the rest of the items being dropped. The scale contains 18 items, which were analyzed individually, rated on a Likert scale, from 0 (not at all) to 10 (very much).

#### 6.2.2. Evaluation of the quality of teaching

Six items were adapted based on The Instructor Evaluation scale (Westfall, 2015). The students were asked to evaluate the teaching process of the teacher in the picture, after listening to the audio sample. Responses were reported on a Likert scale, from 0 (not at all) to 10 (very much). All items were analyzed individually.

#### 6.2.3. Testing students after audition

The test asked students to answer three questions: indicate the main idea of the story, what was the easiest to remember, and what they liked in the story the most. Students could provide open answers to the first two questions, and they reported the answer for the last question on a Likert scale from 0 (not at all) to 10 (very much). The three questions were actually a pretext to provide a grade for each student.

#### 6.2.4. Grade expected by students

It was measured by a single item, and the students were asked to indicate what grade they think they will get after being evaluated by teachers?

In order to evaluate the students’ expectations regarding school grades, the participants in the experiment had to complete a short test regarding the material listened to and the photo viewed. The students were told that the test would be graded from 0 to 10. The test was the first section to be completed, then the research items followed and then the last question asked respondents to rate what grade they thought they would receive on the test.

#### 6.2.5. Student’s openness for more school activities

Four items were created measuring student’s availability for school extra actions. Students were asked whether they would be available to do extra work, listen carefully to lessons, stay extra hours for additional training and whether they liked to teach like this teacher if they were teachers. Responses were indicated on a Likert scale, from 0 (not at all) to 10 (very much). All items were analyzed individually.

#### 6.2.6. The teachers’ age

It was measured by a single item and the respondents evaluated how old they thought the teacher was.

#### 6.2.7. The visual stimuli

They were established according to a pilot study in which several photos were evaluated. Students indicated which photo was more attractive and which photo was the least attractive (see section 6.3). The present study manipulated teachers attractiveness by exposing participants to two types of photos: an attractive photo and an unattractive photo. The study considered female teachers, who were predominant in the Romanian educational system.

#### 6.2.8. Audio recording

It consisted of a 6-min audio recording of a therapeutic story. The recording was played on a laptop that had speakers attached. The recording contained a therapeutic story narrated by a female person with a warm and clear voice, a good, pleasant intonation and a relaxing rhythm to be easily understood by children.

### 6.3. The pilot study

In order to calibrate the measurement tool for evaluating teachers’ attire attractiveness, the method of visual stimuli represented by photographs was selected. Photos were used in previous research methodology (Westfall et al., 2016). The same person was photographed dressed in different clothing. The model is a 24 years old teacher from Suceava, with athletic silhouette and brown, shoulder-length hair cut. For this, a number of 6 clothes considered unattractive and 6 clothes considered attractive were chosen and the model dressed like that. The photos were taken against a white wall, and then the background removed in Canvas. The clothing source was a local clothing shop and the personal wardrobe of the model teacher. The attractiveness or unattractiveness of the photographs was determined, in the first phase, by three research assistants, who discussed and selected the photographs, they representing the group of experts. The overall inter-rater agreement was 92%. The group of experts also decided that there is no need that the face of the persons, being assessed, to be visible, as it would be another parasitic variable and would influence the final results. Therefore the faces of all the models in the pictures were covered in Canvas with a skin-colored oval.

In the pretest phase, each of the 12 photographs was presented to a class of children and they were asked to rate the attractiveness or unattractiveness of each teacher in the photos. The children took part in the pretest phase voluntarily, during classes. Children were asked the following: “*Which teacher is dressed the most attractively?*” and “*Which teacher is dressed the most unattractively?*” The assessment was made on a Likert scale from 0 (not at all attractive) to 10 (very attractive). In accordance with the evaluation of the children, two photos were selected: the most attractive and the least attractive. The independent samples *t*-test indicated that there was a significant difference between the means regarding the attractive model (M = 8.13, SD = 1.03) and the model considered unattractive (M = 4.35, SD = 2.45), *p* < 0.001. The model teacher required the pictures to remain confidential, so they were not published, but the clothing for each model was described.

The pre-test indicated that the most attractive dress pattern for female teachers is *black regular pants and black suit jacket with long sleeves and a white button-down shirt with a narrow high neck collar, with skin-colored nails and cream heels*. Research indicated that there are generally 3 types of clothing studied: (1) business attire (eng. Grooming and business dress attire or business professional), (2) relaxed business attire (eng. Business casual attire), (3) relaxed attire (Eng. casual attire). This pilot study indicated that the most attractive clothing for women is not sexy or eye-catching (Fasoli et al., 2018), but business-friendly and non-color, white and black. This clothing style best conveys the idea of professionalism of teachers, as compared to the other types of clothing (Ruetzler et al., 2012). The students’ evaluation indicated, at least in this Romanian cultural context, that other forms of dressing are less attractive in the school context. They are: (1) Red body-hugging, above-the-knee dress with a slight slit in the front and black heels, (2) yellow, loose, long prom dress, (3) light gray knee-length skirt, sleeveless black blouse with a low neckline, with a discreet belt at the waist and black stilettos, (4) black knee-length skirt with a stiletto in the front and a loose jacket, and underneath a white briefcase and black stilettos, (5) gray suit with knee-length skirt and normal jacket with long sleeves, with a black shirt with white popcorn and black heels.

The students also indicated that the most unattractive outfit is the one *with atypical long black heels, black skirt with cherry fringes and lace, orange leather jacket with belt and neck scarf.* The other five outfits presented to students as unattractive were: (1) slim blue jeans with white faded knees, and the right knee ripped, with white sneakers with black laces, no socks, with a black long-sleeved hoodie with a white print on the left chest and with prints on the exterior sleeves from top to bottom. (2) black sneakers with black laces, no socks, with blue slim jeans and a dark red cotton briefcase with long sleeves and a collar. (3) white glossy one-piece dress with a cream horizontal stripe pattern, and a glossy black shawl stitched over the shoulder fastened with a fringed belt clip, with brown high flat sandals. (4) beltless regular style low flared jeans, with finger long sleeves, rectangle anchor blouse with thick white and black horizontal stripes, glossy black leather heelless loafers, no socks (5) black leather heelless boots with a front buckle, no laces, with regular wide leg blue jeans, with a white collared white shirt with white buttons, with the first two buttons undone as a neckline, with a brown suede jacket with black buttons and long slim sleeves above the wrist.

The unattractive outfit would fall under the casual clothing style (Harbin, 2018). Although the purpose of the pilot study was not to classify teachers’ attire in two categories, based on the clothing indicators, it can be easily concluded that the attractive photo represents the formal business attire, while the unattractive photo is closer to the casual style.

The two images selected by the children, the most attractive and the most unattractive, were retained for use further in the research.

### 6.4. Data collection and statistical analysis strategy

Experimental research data was collected through individual self-reports, based on a questionnaire, applied in classrooms, in schools. The questionnaire was filled up after the participants were exposed to the experimental stimuli.

In order to collect information from participants, the following principles and steps were applied. (1) Each class of students received a photo, which was placed on the table, between the two students. (2) The photo was in A4 format, laminated and color. Every two students sitting down in a desk had assigned one photo. (3) Each class had either the attractive or the unattractive photo, but not both. Researchers manipulated teachers’ attractiveness by exposing respondents to two conditions: attractive teacher condition and unattractive teacher condition. (4) Then the children were informed that they were to listen to a sample lesson, taught by the teacher in the picture, from the desk. In fact, the same audio recording was played for all children; no matter they watched the attractive or the unattractive photo. (5) The audio recording was about 6 min long and was a therapeutic story for children. (6) Respondents were asked not to take any notes during the audition. (7) They were also informed that they would receive a test after listening to the recording, which would be graded. (8) The audio file was played on a computer. (9) After the audition, each student was given a set of questionnaires, which they filled up. The questionnaire contained 2 sections. In the first section there were 5 questions about the content of the audio file, which was actually the lesson delivered by the teacher. The second section contained items necessary for the current research.

The *t*-test for independent samples was used to analyze the data, in SPSS and jamovi.

### 6.5. Hypotheses

There were formulated five hypotheses which were derived based on the scientific literature:

*H1*: Children will be more open to the attractive teacher as compared to the unattractive teacher.

*H2*: Children will attribute more positive personality traits to the more attractive teacher than to the unattractive teacher.

*H3*: Attractive teachers will receive more positive evaluations on their teaching than unattractive teachers.

*H4*: Children will expect a higher grade from the attractive teacher than from the unattractive teacher.

*H5*: Children will perceive the attractive teachers as being younger than unattractive teachers.

### 6.6. Results

In order to check whether children will be more open to the attractive teacher as compared to the unattractive teacher we applied the *t*-test for independent samples, in Jamovi, for each continuous variable (see Tables 1, 2).

**Table 1:** Results for the *t* test: item description, Welch’s *t* test value, degrees of freedom, *p* value and mean difference.

Hypothesis 1
Item	Welch’s *t*-test	df	*p*	Mean difference
If the person you see in the photo were your teacher, how open would you be to:
1. Do extra homework on this subject	8.61	121	<0.001	2.84
2. Listen to her carefully on her subject	6.14	100	<0.001	1.74
3. Stay extra hours, for additional training	3.89	143	<0.001	1.41
4. Teach like this teacher	5.99	130	<0.001	2.44
Hypothesis 2	Welch’s *t*-test	df	*p*	Mean difference
Ambitious	6.60	111.4	<0.001	2.17
Broadminded	5.83	101.6	<0.001	1.83
Capable	5.66	89.8	<0.001	1.61
Cheerful	5.56	88.9	<0.001	1.69
Clean	5.41	100.3	<0.001	1.46
Courageous	5.39	95.7	<0.001	1.48
Forgiving	7.64	95.2	<0.001	2.77
Helpful	7.14	99.4	<0.001	2.62
Honest	6.91	114.1	<0.001	2.06
Imaginative	5.77	99.8	<0.001	1.89
Independent	6.24	131.4	<0.001	2.42
Intellectual	5.08	86.7	<0.001	1.47
Logical	5.96	88.6	<0.001	1.79
Loving	7.24	82.3	<0.001	2.09
Obedient	6.97	91.0	<0.001	2.04
Polite	5.21	102.7	<0.001	1.68
Responsible	5.47	83.5	<0.001	1.55
Self-controlled	5.39	124.4	<0.001	1.54
Hypothesis 3	Welch’s *t*-test	df	*p*	Mean difference
She *clearly* presents the lesson.	6.24	82.3	<0.001	1.51
She is *organized* when she teaches the lesson.	6.87	94.5	<0.001	2.01
*Knows* the subject taught.	5.44	89.9	<0.001	1.30
She *cares* about the students.	5.25	85.7	<0.001	1.45
She grabs my *attention*.	5.97	100.7	<0.001	2.25
The teacher *prepared* well for the lesson.	5.50	98.9	<0.001	1.46
Hypothesis 4	Welch’s *t*-test	df	*p*	Mean difference
1. What grade do you think you will get on the test?	6.62	102	<0.001	1.75
Hypothesis 5	Welch’s *t*-test	df	*p*	Mean difference
1. How old do you think the teacher is?	−10.5	119	<0.001	−11.5

Table created with Microsoft Word, based on the Jamovi output.

**Table 2:** Descriptive statistics based on the hypotheses tested: Items, Independent Variable Levels (Group), Number of Subjects (*N*), Mean, and Standard Deviation (SD).

Hypothesis 1
Items	Group	*N*	Mean	SD
Do extra homework on this subject	Attractive	98	7.91	1.56
	Unattractive	78	5.06	2.57
Listen carefully to her on the subject	Attractive	98	8.46	1.05
	Unattractive	77	6.71	2.32
Stay extra hours, for additional training	Attractive	95	7.73	2.05
	Unattractive	77	6.31	2.60
Teach like this teacher	Attractive	95	7.62	2.11
	Unattractive	76	5.18	3.00
Hypothesis 2				
Items	Group	*N*	Mean	SD
Ambitious	Attractive	93	8.87	1.393
	Unattractive	77	6.70	2.59
Broadminded	Attractive	93	9.25	1.176
	Unattractive	76	7.42	2.52
Capable	Attractive	95	9.49	0.861
	Unattractive	75	7.88	2.35
Cheerful	Attractive	95	9.59	0.831
	Unattractive	77	7.90	2.57
Clean	Attractive	93	9.34	0.994
	Unattractive	76	7.88	2.18
Courageous	Attractive	94	9.29	1.001
	Unattractive	73	7.81	2.17
Forgiving	Attractive	95	9.24	1.218
	Unattractive	76	6.47	2.96
Helpful	Attractive	93	8.90	1.360
	Unattractive	75	6.28	2.94
Honest	Attractive	94	9.28	1.307
	Unattractive	77	7.22	2.33
Imaginative	Attractive	94	9.50	1.225
	Unattractive	75	7.61	2.61
Independent	Attractive	95	8.52	2.015
	Unattractive	77	6.09	2.88
Intellectual	Attractive	96	9.69	0.799
	Unattractive	75	8.21	2.41
Logical	Attractive	96	9.46	0.882
	Unattractive	75	7.67	2.48
Loving	Attractive	95	9.69	0.68
	Unattractive	74	7.61	2.40
Obedient	Attractive	96	9.51	0.8
	Unattractive	77	7.47	2.45
Polite	Attractive	94	9.45	1.215
	Unattractive	77	7.77	2.61
Responsible	Attractive	94	9.61	0.676
	Unattractive	75	8.05	2.38
Self-controlled	Attractive	95	9.13	1.401
	Unattractive	77	7.58	2.17
**Hypothesis 3**				
Item summary	Group	*N*	Mean	SD
She clearly presents the lesson.	Attractive	90	9.53	0.622
	Unattractive	72	8.03	1.97
She is organized when she teaches the lesson.	Attractive	94	9.53	0.936
	Unattractive	77	7.52	2.43
Knows the subject taught.	Attractive	98	9.62	0.681
	Unattractive	77	8.32	2.00
She cares about the students.	Attractive	96	9.72	0.660
	Unattractive	77	8.27	2.34
She grabs my attention.	Attractive	98	9.04	1.464
	Unattractive	75	6.79	3.01
The teacher prepared well for the lesson.	Attractive	97	9.48	0.969
	Unattractive	76	8.03	2.15
**Hypothesis 4**				
Item	Group	*N*	Mean	SD
What grade do you think you will get on the test?	Attractive	97	9.21	1.01
	Unattractive	76	7.46	2.12
**Hypothesis 5**				
Item	Group	*N*	Mean	SD
How old do you think the teacher is?	Attractive	93	26.9	5.06
	Unattractive	77	38.4	8.44

The results of the analysis indicated that the respondents are: more inclined to do extra homework on the subject when the model is perceived to be more attractive (M = 7.91, SD = 1.56), than when she is perceived to be less attractive (M = 5.06, SD = 2, 57), (*p* < 0.001), more inclined to listen more attentively to the teacher when the model is perceived to be more attractive (M = 8.46, SD = 1.05), than when she is perceived to be less attractive (M = 6.71, SD = 2.32), (*p* < 0.001), more likely to stay for extra hours for training, after classes, when the model is perceived to be more attractive (M = 7.73, SD = 2.05), than when she is perceived to be less attractive (M = 6.31, SD = 2.60), (*p* < 0.001), more inclined, if they were teachers, to teach like that teacher when the role model is perceived to be more attractive (M = 7.62, SD = 2.11), than when the teacher is perceived to be less attractive (M = 5.18, SD = 3.00), (*p* < 0.001). The hypothesis is confirmed. Secondly, in order to check whether children perceive the personality traits of attractive teachers as more positive than of unattractive teachers, it was used the *t*-test for independent samples in Jamovi, for each continuous variable of the scale (see Tables 1, 2). The results indicate that in all *t*-test comparisons, in the attractive condition, the perception of personality traits is more positive than in the unattractive condition, and the *p* value is in all 18 situations < 0.001. The hypothesis is thus confirmed. Thirdly, in order to investigate whether attractive teachers will receive more positive evaluations than the unattractive teachers, it was used the *t*-test for independent samples in jamovi (see Tables 1, 2). The results indicated that there were significant differences (*p* < 0.001) in all six situations tested. Descriptive data for the groups compared above are presented in the table below (Table 2). The results indicate that the attractive teachers are more positively evaluated regarding their teaching than the unattractive teachers. The hypothesis is confirmed. Fourthly, in order to test this hypothesis that ‘Children will expect a higher grade from the attractive teacher than from the unattractive teacher.’ we applied the *t*-test for independent samples (see Table 1). The results indicated that there were significant differences between children who perceived their teacher as unattractive (M = 7.46, SD = 2.12) as compared to those who perceived their teacher as attractive (M = 9.21, SD = 1.01), regarding the expected grade (*p* < 0.001), (see Table 2). The hypothesis is confirmed. Finally, in order to analyze the hypothesis that ‘Children will perceive the attractive teachers as being younger than unattractive teachers’ we applied the *t*-test for independent samples. The results indicated that the attractive teacher is considered younger (M = 26.9, SD = 5.06) in comparison to the unattractive model (M = 38.4, SD = 8.44; see Tables 1, 2). The attractive teacher is perceived to be on average 26.9 years old, while the unattractive teacher is perceived to be on average 38.4 years old. The hypothesis is thus confirmed.

## 7. Discussions

The purpose of this research was to investigate the effect of children’s perception of teacher attire attractiveness on variables related to teacher’s personality and professional performance.

A data collection procedure was established and five research hypotheses were tested. In the preliminary analysis, the results indicated that there are significant differences between the two levels of children’s perception regarding the attractiveness of the teacher’s clothing-attractive condition vs. unattractive condition—regarding teacher’s personality, quality of teaching, grade expected by students, students’ openness for more school activities or teacher’s age.

Regarding the *first hypothesis*, it was found that in all four tested situations the children reported that their openness for school activities is higher in the condition of increased attractiveness, than in the case of low attractiveness of the teacher’s clothing. If children perceive the teacher as more attractive, they are more willing to do extra homework, listen more attentively in class, agree to stay after class for additional training or, if they were to become teachers, and would like to teach like that teacher, except when they perceive the teaching staff as less attractive. Existing studies confirm these results. The clothing style of the teaching staff influences the trust in the teacher, the perception of his expertise or the extent to which he is liked by students (Sebastian and Bristow, 2008), which indirectly could contribute to student motivation. Furthermore, a review of the literature indicates that clothing appears to have an effect on behavior in 85.3% of the studies reviewed (Johnson et al., 2008).

In the case of *the second hypothesis*, the results indicated that the students perceive the personality qualities of the teaching staff differently depending on the perception of the clothing attractiveness. In other words, when the teaching staff is perceived as more attractive, all the assessed personality traits are perceived as more pronounced and register higher scores, compared to the situation in which the teaching staff is perceived as less attractive, in all 18 situations. Existing studies indicate that clothing is a form of communication and implies information about status, intelligence, authority or social position (Webster and Driskell, 1983; Storm, 1987; Harbin, 2018). Clothing tells a story and it is impossible not to communicate something positive or negative (Morris, 1977).

Then, regarding the *third hypothesis*, the children evaluated the quality of the teaching act as lower when they perceived the attractiveness of the teacher’s clothing as lower, compared to the situation when their perception of the attractiveness of the teacher’s clothing was higher. Children who found the teacher model to be more attractive in clothing reported that the teacher presents the lesson more clearly, is more organized when teaching the lesson, knows the subject matter better, cares about the students more, captures the students’ attention more, and is even more prepared for the lesson, than in the situation where the children considered the teacher’s model to be more unattractive. Few studies have investigated the students’ perception of the quality of the teaching process according to the attractiveness of the teaching staff’s clothing. Most focused on the effects of clothing styles on student perceptions. Those teachers who have formal professional clothing are perceived as more prepared to teach, more informed and more organized (Gorham et al., 1999), and this type of clothing is also the one investigated in the present research. Moreover, attractive teachers (formally dressed in our study) are perceived as being more competent, as long as the items filled out, refer to teaching abilities. A very recent study found similar results (Oliver et al., 2022). Formally dressed teachers are perceived to be more competent although variables such as successful communication or discipline norms might contribute to variations in teachers’ competency levels.

*The fourth* hypothesis tested indicated that those children who viewed the attractive photo believed they would receive a higher grade on the test (M = 9.21) compared to those children who viewed the unattractive photo (M = 7.46). Unattractive teachers are perceived as more punitive, while attractive teachers are perceived as more non-punitive (Goebel and Cashen, 1979). Attractive teachers are perceived to have a fairer grading procedure than unattractive ones (Tata, 1999). These biases explain the result obtained here.

Regarding the *last* hypothesis tested, the results indicated that in case of the more attractive photo, students rated the teacher as having a younger age (M_years_ = 26.9), than when the students looked at the less attractive photo (M_years_ = 38.4). Therefore, this hypothesis is also confirmed. This result is in agreement with other scientific results that indicate that, in general, there are more positive traits attributed to attractive teachers compared to those perceived as unattractive (Feingold, 1992).

## 8. Implications

What is more, the results of the present study allow us to derive some implications. *Firstly*, our results indicate that children’s perceptions fluctuate to more positive or more negative in accordance with teachers’ attire in the most important aspects of school life: teacher’s personality and age, quality of teaching, grades expected by students or students’ openness for more school activities. The results indicate that the effects are not negligible and teachers’ attire is not just a wearing. Learning is an effortful activity and teachers’ attire can improve perceptions and bring more positivism in the class. *Secondly*, it is not a mistake to reconsider teachers’ dress code in schools and be sensitive to what is beautiful, esthetic and to what serves teaching profession and learning better. To be well-dressed and to care more about personal clothing style means also to serve an educational purpose, as long as personal attire conveys meanings about school atmosphere, teachers’ world and intentions. *Thirdly*, policy makers or local schools might mediatize the results of such studies to teachers who should know well the effects of their attire on students’ perceptions. Such studies are easy applicable to school contexts. Although todays’ society might be reluctant to more control and rules, schools might implement dress guidelines that help teachers adapt and decide by themselves. *Finally*, teachers have a respectable profession and they teach because it is supposed that they love students, their profession and their school subject. Teachers are expected to be good models in all areas of life and teach for the educational outcome, and their attire serves this purpose.

## 9. Conclusion

The purpose of the present research was to investigate how a series of variables (related to performance, personality traits and behaviors of teachers) vary according to the teachers’ attire attractiveness (attractive vs. unattractive). The results indicated that there were significant differences in all conditions evaluated, and all hypotheses were confirmed. The research indicates that attractive clothing plays an important role in human interactions and brings to the forefront, once again, the discussion about the clothing style in the Romanian education system.

One limitation of this research is that the data is self-reported and there is a risk of response desirability. In addition, the sample of the experiment is relatively small, although the differences tested scored highly significant.

Future research should investigate other aspects of teacher attractiveness. Research could also include other variables that lead to a better understanding of the attractiveness phenomenon, such as the gender of the teaching staff, colors, body accessories, style of teaching, face attractiveness or voice characteristics. What is more, future research should also investigate mechanisms by which teachers’ attire influence different psychological realities in students.

## Data availability statement

The raw data supporting the conclusions of this article will be made available by the authors, without undue reservation.

## Ethics statement

The studies involving human participants were reviewed and approved by Aurel Vlaicu University of Arad, Faculty of Educational Sciences, Psychology and Social Work, Center of Research Development and Innovation in Psychology (ID no. 12/01.Feb. 2022). The ethics committee waived the requirement of written informed consent for participation.

## Author contributions

MM, II, AH, and RR: conceptualization, methodology, software, formal analysis, investigation, resources, data curation, writing—original draft preparation, writing—review and editing, visualization, supervision, project administration, funding acquisition. All authors contributed to the article and approved the submitted version.

## Funding

This paper was funded by University of Life Sciences “King Mihai I” from Timisoara and the Research Institute for Biosecurity and Bioengineering Timisoara, Romania. The funding institution had no role in the research or the preparation of the article.

## Conflict of interest

The authors declare that the research was conducted in the absence of any commercial or financial relationships that could be construed as a potential conflict of interest.

## Publisher’s note

All claims expressed in this article are solely those of the authors and do not necessarily represent those of their affiliated organizations, or those of the publisher, the editors and the reviewers. Any product that may be evaluated in this article, or claim that may be made by its manufacturer, is not guaranteed or endorsed by the publisher.
